# The Effects of Back Schools on Non-Specific Back Pain: A Systematic Review and Meta-Analysis

**DOI:** 10.3390/jpm14030272

**Published:** 2024-02-29

**Authors:** Pablo Hernandez-Lucas, Raquel Leirós-Rodríguez, Juan Lopez-Barreiro, José L. García-Soidán

**Affiliations:** 1 Faculty of Physiotherapy, University of Vigo, Campus A Xunqueira, 36005 Pontevedra, Spain; 2 SALBIS Research Group, Nursing and Physical Therapy Department, University of Leon, Astorga Ave., 24401 Ponferrada, Spain; rleir@unileon.es; 3 Faculty of Education and Sport Sciences, University of Vigo, Campus A Xunqueira, 36005 Pontevedra, Spain; juan.lopez.barreiro@uvigo.es (J.L.-B.); jlsoidan@uvigo.es (J.L.G.-S.)

**Keywords:** musculoskeletal pain, exercise therapy, health education, physiotherapy

## Abstract

Background: Non-specific back pain is a global concern. Exercise and health education are crucial components in its management. The Back School is a theoretical practical program that integrates both elements. The objective of this study is to determine if Back School-based programs are effective in reducing pain, disability, and kinesiophobia in patients with non-specific back pain. Methods: A systematic review of research involving participants with non-specific back pain was carried out on databases such as PubMed, Scopus, Web of Science, and Medline. Results: In total, 25 papers were chosen for review. All of these papers focused on the effects on the lumbar area, with the exception of one paper that specifically targeted the cervical region. The pain variable showed statistically significant results with standardized mean differences of −1.01 (950 confidence interval = −1.39 to −0.63; *p* < 0.001), and the disability variable had standardized mean differences of −0.98 (95% confidence interval = −1.38 to −0.58; *p* < 0.001), and only one study analysed the kinesiophobia variable and concluded that Back School programs have a positive effect on kinesiophobia between the baseline and post-intervention levels. Conclusions: Back School programs have shown effectiveness in reducing non-specific back pain and lowering disability rates.

## 1. Introduction

Back pain is a prevalent and disabling issue, and it is the leading cause of years lived with disability in the world [1]. Non-specific back pain (NSBP) is a common ailment that often leads individuals to seek healthcare services in developed nations [2,3,4]. It plays a significant role in work-related disabilities and imposes a substantial economic burden on society [2,3,4]. In the United States, the aggregate cost soars past USD 97.4 billion, while in the United Kingdom, it approximates around EUR 11 billion [2,3,4]. The most recurrent spinal condition is NSBP [3]. NSBP refers to a condition characterized by pain in the cervical, dorsal, or lumbar region, or a combination of these areas, where the exact cause or underlying disease cannot be identified. It excludes specific conditions like cancer, infection, ankylosing spondylitis, and other inflammatory or infectious conditions [3,5]. NSBP may be associated with modifiable risk factors that encompass personal, social, and occupational aspects [6,7,8,9], such as physical inactivity and catastrophic beliefs about the origin of back pain [7,8,9,10]. Identifying these factors is highly valuable for developing programs aimed at enhancing the management of this condition [10,11]. For this reason, many professionals prescribe theoretical–practical programs, such as Back School, which aims to alleviate and prevent back pain through the implementation of exercises and educational interventions. Theoretically, Back School programs (BSPs) transmit recommendations on healthy lifestyles and information on the erroneous catastrophic beliefs about back pain [12]. In addition, practically, patients are taught to perform strengthening and flexibility exercises for the back musculature [13].

Previous reviews have focused solely on the effects of BSPs on the lumbar region [14,15,16]. The latest review, published in 2017, determined that existing research on the effects of BSPs on the lumbar region has low methodological quality [17]. It emphasized the necessity for additional scientific studies that analyse new variants and aspects of the intervention [17]. To date, no subsequent reviews have been conducted on the effects of BSPs, and no meta-analysis has specifically analysed their impact on NSBP. Despite the widespread application of these programs by various professionals, there is a notable absence of comprehensive studies addressing the significant socioeconomic impact of NSBP globally. Hence, this systematic review with a meta-analysis aimed to assess the impact of BSPs on pain, disability, and kinesiophobia in individuals with NSBP.

## 2. Materials and Methods

### 2.1. Design

This research was registered with PROSPERO (registration code: CRD42023412321) and adhered to the Preferred Reporting Items for Systematic Reviews and Meta-Analyses (PRISMA) [18] guidelines for Exercise, Rehabilitation, Sport Medicine, and Sports (PERSIST) [19], as well as the Cochrane Collaboration’s recommendations [20]. The PICOS question was formulated as follows: P—population: NSBP patients; I—intervention: Back School; C—control: alternative therapeutic intervention, placebo, or no intervention; O—outcome: pain, kinesiophobia, or disability; S—study designs: randomized controlled trial. In March 2023, a systematic investigation was performed utilizing the following databases: PubMed, Scopus, Web of Science and Medline. The search methodology utilized a variety of combinations of the subsequent Medical Subject Headings (MeSH) terms: Back Pain, Low Back Pain, Neck Pain, and Musculoskeletal Pain. The term “Back School” was used as a free term. We also used the filter of each database for the selection of clinical articles. The Supplementary Material’s Table S1 illustrates the search strategy, which is based on the concentrated PICOS question.

### 2.2. Study Selection

Duplicate articles were first removed, after which two independent reviewers (P.H.-L.; J.L.-B.) screened the remaining articles for suitability. In cases where the reviewers disagreed, a third reviewer (R.L.-R.) was consulted to make the final decision on whether to include or exclude a study from the analysis. This method was used to ensure an unbiased and fair evaluation of the studies. The criteria for inclusion in the study selection were as follows: (i) the intervention group in the studies performed BSPs and (ii) the participants in the sample had NSBP. On the other hand, the following studies were excluded from this review: (i) non-randomized controlled trials; (ii) participants with specific causes of back pain; (iii) pregnant women; and (iv) studies where the full text was not available.

After the initial screening of data, the titles and abstracts were evaluated based on the inclusion criteria. The full texts of the abstracts that met these criteria were then obtained. If the titles and abstracts did not provide sufficient information to determine compliance with the inclusion criteria, their full texts were also retrieved. The selection of full-text articles was based on their adherence to the inclusion criteria, as determined by two reviewers using a data extraction form. These two reviewers independently extracted data from the included studies using a custom data extraction spreadsheet in Microsoft Excel. In the case of any disagreements, the reviewers had a discussion until they reached a consensus.

### 2.3. Data Extraction

Data extraction for further analysis was conducted by two reviewers (P.H.-L.; J.L.-B.), encompassing demographic details (title, authors, journal, and year), sample attributes (age, gender, and participant count), and specific study parameters (intervention duration, adverse events, exercise methodologies, and health education), along with the outcomes (analysed variables, instruments employed, and follow-up duration). Tables were employed to present the characteristics of the studies and the extracted data.

### 2.4. Quality Assessment

The studies’ quality was assessed using the PEDro scale, and the RoB (Risk of Bias) tool was used to evaluate the risk of bias. The GRADE system was implemented to ascertain the overall certainty of the evidence. These assessments were conducted by two reviewers (P.H.-L. and J.L.-B.) using the PEDro scales, the RoB tool, and the GRADE system. Should there be any disagreement, a third author (R.L.-R.) was involved in the deliberation process to achieve consensus.

### 2.5. Data Analysis

The calculation of standardized mean differences, along with their 95% confidence intervals, was achieved by dividing the mean difference between groups by the combined standard deviation [21]. If such information was not available in the study, the authors were contacted via email to gather the necessary data. The interpretation of effect sizes followed predefined cut-off values ranging from 0 to 0.2 for a very small effect; from 0.2 to 0.5 for a small effect; from 0.5 to 0.8 for a moderate effect; and anything above 0.8 for a strong effect [22]. A significance level was set at *p* < 0.05. The I^2^ statistic was used to measure the levels of heterogeneity, with the percentages representing the degree of heterogeneity: 25% for low, 50% for medium, and 75% for high heterogeneity [21]. Due to the detected heterogeneity, a random-effects model was employed for the meta-analysis. The analyses were performed using the Comprehensive Meta-Analysis (CMA) V2 software by Biostat, Englewood, NJ, USA.

## 3. Results

### 3.1. Flow of Studies through the Review

**Figure 1:**
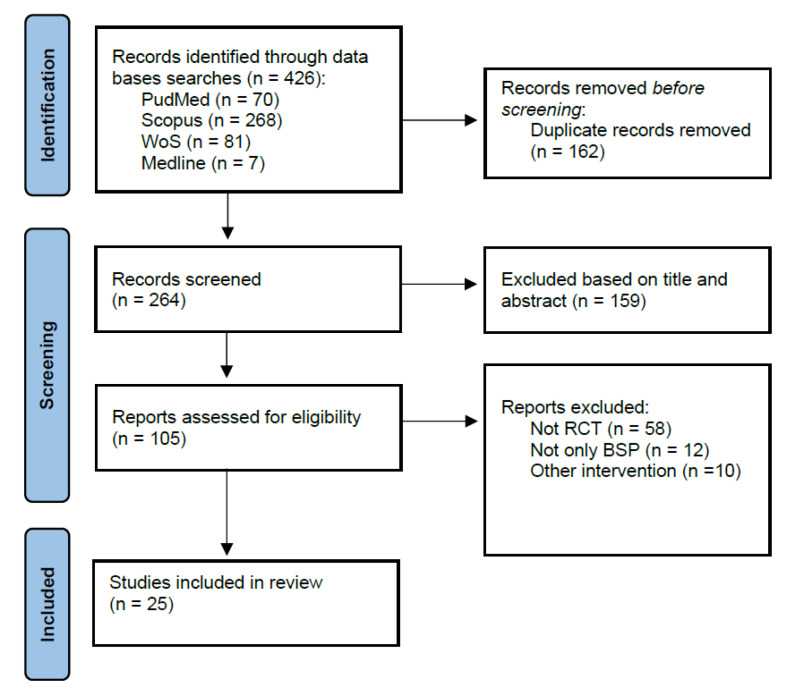
PRISMA flow diagram.

From a total of 426 search results, 264 studies were deemed suitable for inclusion after the removal of duplicates. Out of these 264 screened papers, 159 were discarded following the screening of the titles and abstracts. Upon the initial reading of all potential full texts, the Kappa score for the first and second reviewers was 0.9, indicating near-perfect agreement [23]. All of the 25 full-text articles that were evaluated for eligibility were ultimately incorporated into the synthesis [24,25,26,27,28,29,30,31,32,33,34,35,36,37,38,39,40,41,42,43,44,45,46,47,48] (Figure 1).

### 3.2. Methodological Quality of the Studies

When assessing the methodological quality using the PEDro scale, all studies achieved a score of five or more, with the exception of three articles [29,40,48] that scored four points. The most variable item on the PEDro scale was the blinding of subjects [24,25,26,28,29,30,31,32,33,34,35,36,37,38,39,40,41,42,43,44,45,47,48], therapists [24,26,28,29,30,31,32,33,34,35,36,37,38,39,40,41,42,43,44,45,46,47,48], and assessors [24,26,27,28,29,30,32,33,34,35,36,37,38,39,40,41,44,45,48], as well as the lack of an intention-to-treat analysis [24,25,27,28,29,30,32,34,35,36,37,38,39,40,41,42,44,45,47,48]. The average score for all articles assessed using the PEDro scale was 5.9 points, which was deemed to be of average methodological quality [49] (Table 1).

### 3.3. Risk of Bias

**Figure 2:**
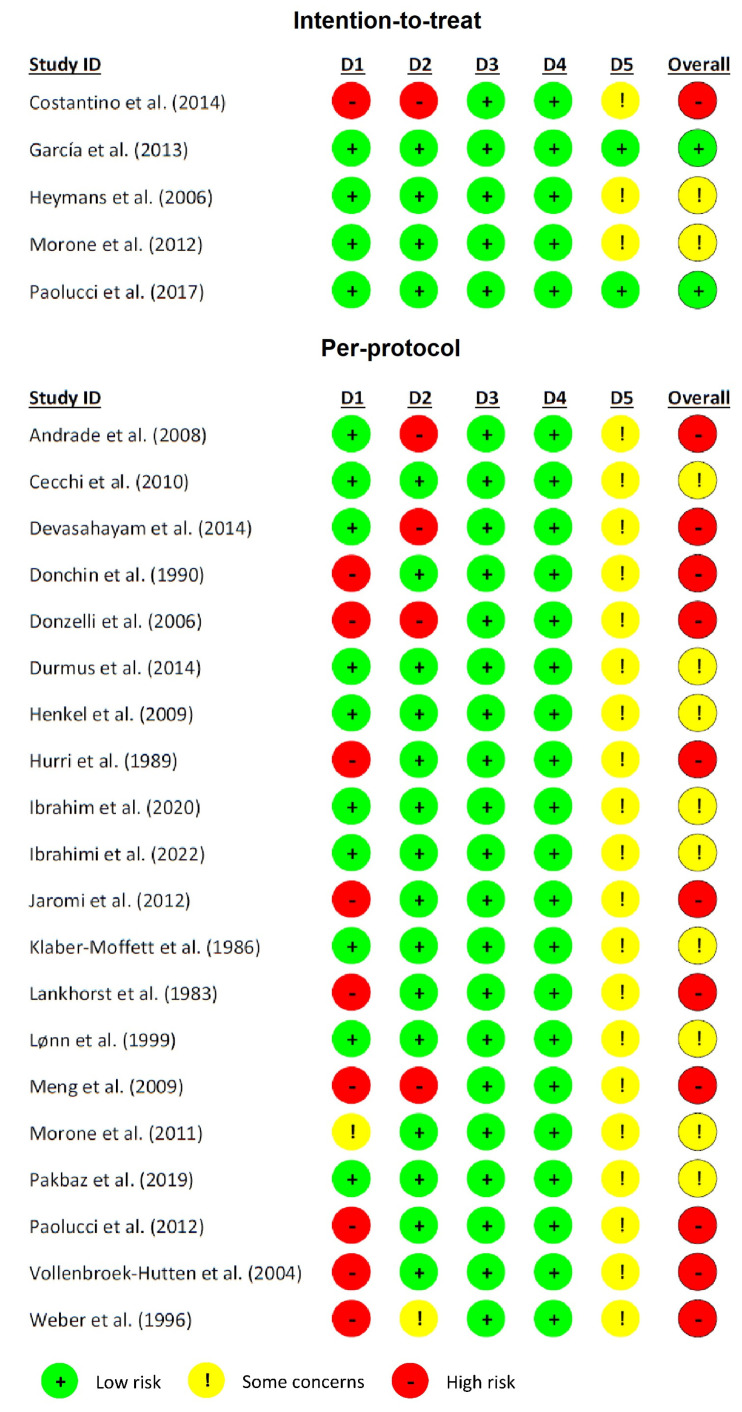
Risk of Bias. D1: process of random assignment; D2: discrepancies from the planned interventions; D3: absence of outcome data; D4: outcome measurement; D5: choice of the result that is reported [24,25,26,27,28,29,30,31,32,33,34,35,36,37,38,39,40,41,42,43,44,45,46,47,48].

In our evaluation of bias, we adhered to the guidelines provided by the Cochrane Collaboration [20]. This was applied to the 25 studies that were included in our analysis [24,25,26,27,28,29,30,31,32,33,34,35,36,37,38,39,40,41,42,43,44,45,46,47,48], and 10 were evaluated with a high risk of bias [26,28,29,34,37,38,40,45,47,48]. In one of them, the randomization method was not indicated, so they were indicated with an uncertain risk of bias [42]. The other 14 included studies that performed the correct randomization, so they were labelled as studies with a low risk of bias [24,25,27,30,31,32,33,35,36,39,41,43,44,46]. In relation to bias due to deviations from intended interventions, five of the included papers presented a high risk of bias in this section [24,26,27,29,40]. Only one was assessed to have an uncertain risk of bias [48]. The other 19 studies included in this review were declared to have a low risk of bias [25,28,30,31,32,33,34,35,36,37,38,39,41,42,43,44,45,46,47]. Regarding bias due to missing outcome data and the risk of bias in the measurement of the outcome, all of the studies included in this systematic review obtained a low risk of bias in the assessment [24,25,26,27,28,29,30,31,32,33,34,35,36,37,38,39,40,41,42,43,44,45,46,47,48]. Lastly, regarding the bias that could occur in the choice of the result to be communicated, 23 papers were rated with an uncertain risk of bias [24,25,26,27,28,29,30,32,33,34,35,36,37,38,39,40,41,42,43,44,45,47,48]. Two of the studies assessed in this review obtained a rating denoting a low risk of bias [31,46] (Figure 2).

#### 3.3.1. Assessment of Certainty

The GRADE system’s assessment indicates a moderate level of certainty. This means that there is a possibility that future research will modify the current understanding of the effects assessed and change the estimate [50] (Table 2).

#### 3.3.2. Participants

The 25 studies involved a total of 4454 participants suffering from NSBP. The participants’ average age was 49.9 ± 9.5 years, and women constituted 62.2% of the sample (Table 3). Notably, seven papers [24,27,28,29,32,46,47] did not disclose the female percentage. All participants had non-specific low back pain, except for those in Henkel et al.’s study [32], who had non-specific neck pain. Adverse effects were only reported in the study by Heymans et al. [33], where three participants from the BSP groups and four from the usual care group experienced a significant increase in low back pain.

### 3.4. Interventions

On average, the studies conducted 10.7 ± 8.9 sessions, with 4 [24,31,33,36,38], 10 [29,42,43,45,46], and 24 [26,28,32] sessions being the most common per intervention. The mean session duration was 62.9 ± 21.6 min, with the majority of sessions lasting between 45 and 60 min [24,25,26,27,28,29,30,31,33,34,37,38,39,40,42,43,45,46]. The duration of the sessions was not described in three articles [32,38,41]. The interventions typically lasted for an average of 5.9 ± 3.5 weeks, with 4 weeks being the most common duration [24,27,29,31,36,42,43,45]. (see Table 3 for more information).

The exercise interventions typically included a warm-up, main part, and cool-down. Strength and stretch trunk exercises [24,25,26,27,28,30,31,33,35,39,41,42,43,44,45,46,48] were the most frequently performed exercises. The most discussed educational topics were ergonomics [24,25,26,27,28,30,31,35,42,43,44,48], the origins and causes of pain [25,26,37,40,42], and cognitive behavioural therapy [27,33].

The focus of all of the articles was the treatment of the lumbar region, except for the study by Henkel et al. [32], which analysed the effect on the cervical region [32]. These interventions were supervised by physiotherapists in all of the studies, except in the study by Ibrahimi et al. [36], which was supervised by an occupational therapist. However, it is worth mentioning that some interventions also involved physicians [25,26,29,35,39,40,41,43,46], occupational therapists [33,35,36], psychologists [40,47], chiropractors [39], or trainers [47]. More information can be found in Table S2 of the Supplementary Materials.

### 3.5. Effects on Pain, Disability, and Kinesiophobia

**Figure 3:**
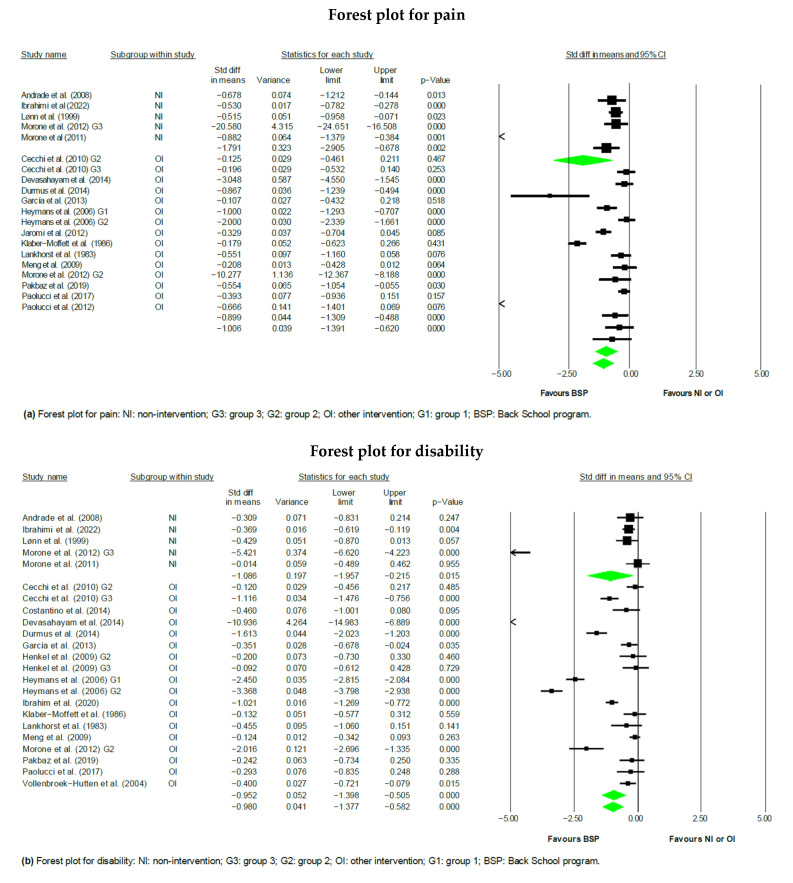
Forest plots: (**a**) Forest plot for pain [24,25,27,30,31,33,36,37,38,39,40,41,42,43,44,45,46] and (**b**) Forest plot for disability [24,25,26,27,29,30,31,32,33,35,36,38,39,40,41,42,43,44,46,47].

In the studies examined, 20 of them assessed the variable of pain [24,25,27,29,30,31,33,34,36,37,38,39,40,41,42,43,44,45,46,48], with 17 being incorporated into the meta-analysis [24,25,27,30,31,33,36,37,38,39,40,41,42,43,44,45,46]. The meta-analysis was split into two categories: BSP versus other therapeutic interventions or no intervention. The first subgroup’s meta-analysis showed a statistically significant reduction in the pain score for the BSP group compared to the non-intervention group, with an SMD of −1.79 (95% CI = −2.91 to −0.68; *p* < 0.01; I^2^ = 95.75%). Additionally, the second subgroup’s analysis showed a statistically significant reduction in the pain score for the BSP group compared to the other therapeutic intervention group, with an SMD of −0.90 (95% CI = −1.31 to −0.49; *p* < 0.001; I^2^ = 93.25%). Ultimately, the results showed a statistically significant reduction in the pain score for the overall BSP group compared to the other therapeutic intervention or non-intervention groups, with an SMD of −1.01 (95% CI = −1.39 to −0.63; *p* < 0.001; I^2^ = 93.71%) (Figure 3).

In the studies analysed, 20 papers [24,25,26,27,29,30,31,32,33,35,36,38,39,40,41,42,43,44,46,47] assessed the disability variable, with 19 being included in the meta-analysis [24,25,26,27,30,31,32,33,35,36,38,39,40,41,42,43,44,46,47]. The meta-analysis was split into two categories: BSP versus other therapeutic interventions or no intervention. The first subgroup’s meta-analysis showed a statistically significant reduction in the disability score for the BSP group compared to the non-intervention group, with an SMD of −1.09 (95% CI = −1.96 to −0.22; *p* < 0.05; I^2^ = 94.29%). Additionally, the second subgroup’s analysis showed a statistically significant reduction in the disability score for the BSP group compared to the other therapeutic intervention group, with an SMD of −0.95 (95% CI = −1.40 to −0.51; *p* < 0.001; I^2^ = 95.41%). Ultimately, the results showed a statistically significant reduction in the disability score for the overall BSP group compared to the other therapeutic intervention or non-intervention groups, with an SMD of −0.98 (95% CI = −1.38 to −0.58; *p* < 0.001; I^2^ = 95.12%) (Figure 3).

Lastly, regarding the impact on kinesiophobia, only Heymans et al. [33] evaluated this in their research. They concluded that the BSP had a beneficial effect on kinesiophobia, as measured by the Tampa Scale of Kinesiophobia, between the baseline and post-intervention levels [33].

### 3.6. Risk of Publication Bias

**Figure 4:**
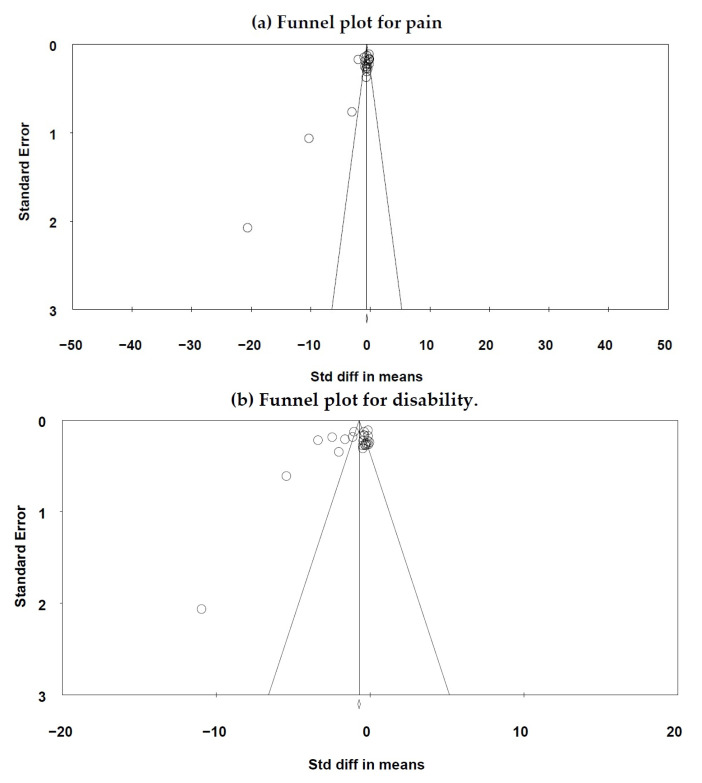
(**a**) Funnel plot for pain; (**b**) Funnel plot for disability.

The funnel plot for referring to pain reduction shows a suggestion of publication bias (Figure 4). Egger’s test for a regression intercept gave *p* = 0.001 and *p* < 0.01, and as these values are lower than 0.10, they indicate possible publication bias [51]. Also, the funnel plot for referring to disability reduction shows a suggestion of publication bias (Figure 4). Egger’s test for a regression intercept gave *p* = 0.098, and as it is lower than 0.10, it indicates possible publication bias [51].

## 4. Discussion

The aim of this research was to examine the impact of BSP on patients with NSBP, focusing on pain, disability, and kinesiophobia. The findings indicate that the BSP has a positive influence on NSBP. The pain variable in these studies [24,25,27,30,31,33,36,37,38,39,40,41,42,43,44,45,46] was strongly affected by the BSP. Various reviews have validated the advantages of physical activity in treating NSBP in both the lumbar [52,53,54,55] and cervical areas [56,57,58,59]. However, the beneficial impact of education on NSBP is still uncertain. The outcomes appear to suggest that interventions combining theory and practice yield better results in treating NSBP than interventions that are solely practical or theoretical. This effect might be attributed to the multifaceted nature of NSBP: some risk factors for NSBP are biophysical, such as a lack of strength or flexibility in the spinal muscles [60,61]; others are psychological, such as fear or stress; and there are even social factors, such as misconceptions about NSBP or work-related factors [8,62]. A prior review of the effects of BSPs on the lumbar region also yielded positive results [63]. There is moderate evidence suggesting that a BSP, in a work setting, reduces pain and improves function and the return-to-work status compared with exercises, manipulation, myofascial therapy, advice, placebo, or waiting-list controls for patients with chronic and recurrent low back pain [63]. However, another review that includes studies up to 2016 did not find such beneficial effects; this may be due to the shift in the current paradigm of NSBP treatment, where the biopsychosocial model is advocated [64].

The variable of disability demonstrated a strong impact in the meta-analysis [24,25,26,27,30,31,32,33,35,36,38,39,40,41,42,43,44,46,47]. This outcome is in line with expectations, reflecting the strong association between disability and pain [65]. Essentially, this correlation stems from the interplay between physical aspects (like neural activation) and psychosocial factors (such as motivation) [65]. In line with this, Frizziero et al. [53] reported the perceived benefits from exercise in the lumbar area, and similar observations were made for the cervical region [58]. Additionally, disability is related to kinesiophobia [66]. Specifically, individuals with NSBP and elevated kinesiophobia levels are 41% more likely to experience disability [66]. This link is likely due to the pivotal role of graded exercise and patient education in mitigating kinesiophobia [67]. Indeed, the International Association for the Study of Pain emphasizes the interconnectedness of fear, pain, and knowledge [68]. This suggests that pain transcends being just a physical sensation [68]. It is also an emotional experience that can be influenced by various emotions, such as anxiety or fear of the unknown [68].

The most recent clinical intervention guideline for NSBP, issued in 2021, highlights exercise and education as fundamental components of NSBP management [69].

Significantly, this is the inaugural meta-analysis that investigates the effects of BSPs on pain across all regions of the spine.

The authors acknowledge that the limitations of this study include extremely high heterogeneity and possible publication bias. It is also crucial to highlight the scarcity of studies investigating the impact of BSP on the cervical and dorsal regions, as well as the exploration of the kinesiophobia variable. Therefore, further studies are recommended, especially in the cervical and dorsal areas, to evaluate the outcomes of various interventions. This will help to develop specific treatment protocols for NSBP.

## 5. Conclusions

BSPs could mitigate pain and lower disability rates among patients with NSBP. Nonetheless, it is essential to further investigate the effects of BSPs, especially in kinesiophobia and in the cervical and dorsal back regions. The findings could enable healthcare workers to enhance the efficacy of their clinical procedures, thereby diminishing the significant socio-economic burden posed by NSBP globally.

## Figures and Tables

**Table 1:** Evaluation of bias risk using the PEDro scale from the Physiotherapy Evidence Database.

Author	1 *	2	3	4	5	6	7	8	9	10	11	Score
Andrade et al. (2008) [24]	✓	✓	✓	✓	✗	✗	✗	✗	✗	✓	✓	5
Cecchi et al. (2010) [25]	✓	✓	✓	✓	✓	✗	✓	✓	✗	✓	✓	8
Costantino et al. (2014) [26]	✓	✓	✗	✓	✗	✗	✗	✓	✓	✓	✓	6
Devasahayam et al. (2014) [27]	✓	✓	✓	✓	✓	✓	✗	✗	✗	✓	✓	7
Donchin et al. (1990) [28]	✓	✓	✗	✓	✗	✗	✗	✓	✗	✓	✓	5
Donzelli et al. (2006) [29]	✓	✓	✗	✓	✗	✗	✗	✗	✗	✓	✓	4
Durmus et al. (2014) [30]	✓	✓	✓	✓	✗	✗	✗	✓	✗	✓	✓	6
García et al. (2013) [31]	✓	✓	✓	✓	✗	✗	✓	✓	✓	✓	✓	8
Henkel et al. (2009) [32]	✓	✓	✓	✓	✗	✗	✗	✓	✗	✓	✓	6
Heymans et al. (2006) [33]	✓	✓	✓	✓	✗	✗	✗	✓	✓	✓	✓	7
Hurri et al. (1989) [34]	✓	✓	✗	✓	✗	✗	✗	✓	✗	✓	✓	5
Ibrahim et al. (2020) [35]	✓	✓	✓	✓	✗	✗	✗	✓	✗	✓	✓	6
Ibrahimi et al. (2022) [36]	✓	✓	✓	✓	✗	✗	✗	✓	✗	✓	✓	6
Jaromi et al. (2012) [37]	✓	✓	✗	✓	✗	✗	✗	✓	✗	✓	✓	5
Klaber-Moffett et al. (1986) [41]	✓	✓	✓	✓	✗	✗	✗	✓	✗	✓	✓	6
Lankhorst et al. (1983) [38]	✓	✓	✗	✓	✗	✗	✗	✓	✗	✓	✓	5
Lønn et al. (1999) [39]	✓	✓	✗	✓	✗	✗	✗	✓	✗	✓	✓	5
Meng et al. (2009) [40]	✓	✓	✗	✓	✗	✗	✗	✗	✗	✓	✓	4
Morone et al. (2012) [43]	✓	✓	✓	✓	✗	✗	✓	✓	✓	✓	✓	8
Morone et al. (2011) [42]	✓	✓	✓	✓	✗	✗	✓	✓	✗	✓	✓	6
Pakbaz et al. (2019) [44]	✓	✓	✓	✓	✗	✗	✗	✓	✗	✓	✓	6
Paolucci et al. (2017) [46]	✓	✓	✓	✓	✓	✓	✓	✓	✓	✓	✓	9
Paolucci et al. (2012) [45]	✓	✓	✗	✓	✗	✗	✗	✓	✗	✓	✓	5
Vollenbroek-Hutten et al. (2004) [47]	✓	✓	✗	✓	✗	✗	✓	✓	✗	✓	✓	6
Weber et al. (1996) [48]	✓	✓	✗	✓	✗	✗	✗	✗	✗	✓	✓	4

The following are the standards: (1) the eligibility requirements are clearly defined; (2) participants were randomly assigned to different groups; (3) the allocation was kept hidden; (4) the groups were comparable at the start; (5) all participants were blinded; (6) all therapists were blinded; (7) all evaluators were blinded; (8) data were collected from over 85% of the participants assigned to groups; (9) participants were given the treatment or control condition as assigned, or an intention-to-treat analysis was conducted; (10) statistical comparisons between groups were reported for at least one outcome; (11) both point estimates and variability measures were provided. High signifies a high risk of bias, while low signifies a low risk of bias. * Pertains to external validity and does not contribute to the overall score; ✓: yes; ✗: not.

**Table 2:** Certainty of the evidence (GRADE).

Outcomes	No. of Participants (Studies)	Risk of Bias	Inconsistency	Indirectness	Imprecision	Other Considerations	Absolute Effect	Certainty of the Evidence
Pain	3718 (20 RCTs)	not serious ^a^	Serious ^b^	not serious	Serious ^d^	publication bias strongly suspected very strong association ^c,e,f,g^	SMD −1.01 [−1.30 to −0.63]	⨁⨁⨁◯ Moderate
Disability	2602 (20 RCTs)	not serious ^a^	Serious ^b^	not serious	Serious ^d^	publication bias strongly suspected very strong association ^c,e,f,g^	SMD −0.98 [−1.38 to −0.58]	⨁⨁⨁◯ Moderate

SMD: standardized mean difference. ⨁⨁⨁◯; moderate; ^a^ The average methodological quality of the studies according to the PEDro scale is good. ^b^ Low methodological heterogeneity but high statistical heterogeneity among trials (I^2^ > 25%). ^c^ The Funnel Plot diagram shows possible publication bias and the Egger’s test shows *p* < 0.10. ^d^ The confidence interval is small, but not all articles calculate the optimal sample size. ^e^ SMD of 0.8 or higher is considered a very large effect. ^f^ The influence of all plausible residual confounding factors is not considered. ^g^ There is no evidence of a dose–response gradient considering the number of doses in the sessions.

**Table 3:** Characteristics of the studies.

Authors	Pain Area	Initial Sample (Women) Mean Age	Intervention (Final Sample)	Supervisor	Weeks	Total Sessions (Duration)	Outcome Measures	Results
Andrade et al. (2008) [24]	NLBP	70 (Not described) 45	G1 (*n* = 29): BSP G2 (*n* = 28): NI	PT	4	G1: 4 sessions (60′)	VAS; RMDQ; SCHOBER	There was a significant enhancement in all results for G1 from the initial stage to the point after the intervention. G2 did not significantly improve from baseline to post-intervention in any outcomes.
Cecchi et al. (2010) [25]	NLBP	210 (67%) 59	G1 (*n* = 68): BSP G2 (*n* = 68): Individual physiotherapy G3 (*n* = 69): Spine manipulation	PT; PH	3	G1: 15 sessions (60′) G2: 15 sessions (60′) G3: 4–6 sessions (20′)	PRS; RMDQ	There was a significant enhancement in all results for all groups from the initial stage to the point after the intervention. G3 significantly improved in all outcomes at post-intervention versus G1 and G2. There were no significant differences at post-intervention between G1 and G2.
Costantino et al. (2014) [26]	NLBP	54 (44.4%) 73	G1 (*n* = 27): BSP G2 (*n* = 27): Hydrotherapy	PT; PH	12	G1: 24 sessions (60′) G2: 24 sessions (60′)	RMDQ; SF-36	There was a significant enhancement in all results for G1 and G2 from the initial stage to the point after the intervention. There were no significant differences at post-intervention between G1 and G2.
Devasahayam et al. (2014) [27]	NLBP	28 (Not described) 54	G1 (*n* = 9): BSP G2 (*n* = 6): ET	PT	4	G1: 1 session (60′) G2: 1 session (60′)	NSR; RMDQ; GPE; PSFS	There was a significant enhancement in all results for G1 from the initial stage to the point after the intervention. G2 significantly improved function from baseline to post-intervention.
Donchin et al. (1990) [28]	NLBP	142 (Not described) 46	G1 (*n* = 46): BSP G2 (*n* = 46): Calisthenics for the back G3 (*n* = 50): NI	PT	G1: 10 G2: 12	G1: 5 sessions (90′) G2: 24 sessions (45′)	Flexion and extension ROM of the back; Kraus-Weber strength test	G2 significantly improved trunk flexion strength and flexibility over the other groups. G2 significantly improved in extension ROM from baseline to post-intervention but without any significant differences between groups.
Donzelli et al. (2006) [29]	NLBP	53 (Not described) 50	G1 (*n* = 22): BSP G2 (*n* = 21): Pilates	PT; PH	4	G1: 10 sessions (60′) G2: 10 sessions (60′)	SPP; ODI	There was a significant enhancement in all results for both groups from the initial stage to the point after the intervention. There were no significant differences between groups.
Durmus et al. (2014) [30]	NLBP	121 (100%) 53	G1 (*n* = 61): BSP G2 (*n* = 60): ET	PT	12	G1: 36 sessions (60′) G2: 36 sessions (60′)	VAS; ODI; SF-36; 6MWT; BDI; FFD; Schober; TFMS; TEMS; QMS	There was a significant enhancement in all results for both groups from the initial stage to the point after the intervention. G1 significantly improved in all outcomes except mobility at post-intervention versus G2.
García et al. (2013) [31]	NLBP	148 (73.6%) 54	G1 (*n* = 72): BSP G2 (*n* = 74): McKenzie	PT	4	G1: 4 sessions (60′) G2: 4 sessions (60′)	NRS; RMDQ; Flexion ROM; WHOQOL-BREF	Both groups significantly improved in all outcomes from baseline to post-intervention. G2 significantly improved versus G1 in disability. There were no significant differences between groups in pain, flexion ROM, or quality of life.
Henkel et al. (2009) [32]	NNP	93 (Not described) 51	G1 (*n* = 27): BSP G2 (*n* = 28): Nordic Walking G3 (*n* = 30): Barefoot Walking	PT	12	G1: 24 sessions (Not described) G2: 24 sessions (Not described) G3: 12 sessions (Not described)	EuroQol-5D; NDI; FFbH; SF-36	There was a significant enhancement in all results for G1 from the initial stage to the point after the intervention. G2 significantly improved from baseline to post-intervention in all outcomes except quality of life and mental component summary of SF-36. G3 significantly improved from baseline to post-intervention in all outcomes except SF-36.
Heymans et al. (2006) [33]	NLBP	299 (21.1%) 40	G1 (*n* = 98): BSP high intensity G2 (*n* = 98): BSP low intensity G3 (*n* = 103): UC	PT; PH; occupational therapist	G1: 8 G2: 4	G1: 16 sessions (60′) G2: 4 sessions (120′)	VAS; RMDQ; TSK; days of sick leave; perceived recovery	All groups improved in all outcomes from baseline to post-intervention. There were no significant differences between groups.
Hurri (1989) [34]	NLBP	204 (100%) 46	G1 (*n* = 93): BSP G2 (*n* = 92): NI	PT	3	G1: 3 sessions (60′) + 1 session (120′)	VAS; LBP index	There was a significant enhancement in pain for G1 versus G2.
Ibrahim et al. (2020) [35]	NLBP	284 (100%) Not described	G1 (*n* = 140): BSP G2 (*n* = 141): UC	PT; PH; occupational therapist	6	G1: 13 sessions (120′)	ODI	There was a significant enhancement in disability for G1 from baseline to third and sixth week. G2 significantly improved disability from baseline to sixth week. There was a significant enhancement in disability for G1 versus G2.
Ibrahimi et al. (2022) [36]	NLBP	250 (0%) Not described	G1 (*n* = 125): BSP G2 (*n* = 125): NI	Occupational therapist	4	G1: 4 sessions (120′)	VAS; RMDQ, SF-36	There was a significant enhancement in all results for G1 from the initial stage to the point after the intervention. G1 significantly improved in both outcomes versus G2.
Jaromi et al. (2012) [37]	NLBP	111 (16.2%) 32	G1 (*n* = 56): BSP G2 (*n* = 55): Passive physiotherapy	PT	6	G1: 6 sessions (50′) G2: 6 sessions (Not described)	VAS; ZEBRIS	There was a significant enhancement in all results for both groups from the initial stage to the point after the intervention. G1 significantly improved from baseline to post-intervention in body posture.
Klaber-Moffett et al. (1986) [41]	NLBP	78 (50%) 41	G1 (*n* = 40): BSP G2 (*n* = 38): ET	PT; PH	3	G1: 3 sessions (Not described) G2: 3 sessions (Not described)	VAS; ODI	There was not a significant enhancement in all results in any group from the initial stage to the point after the intervention. G1 significantly improved in body posture from the initial stage to the point after the intervention.
Lankhorst et al. (1983) [38]	NLBP	48 (56%) 51	G1 (*n* = 21): BSP G2 (*n* = 22): Electrotherapy	PT	2	G1: 4 sessions (45′) G2: 4 sessions (Not described)	VAS; MFCS	There was a significant worsening in all results for G2 from the initial stage to the point after the intervention. There were no significant differences between groups.
Lønn et al. (1999) [39]	NLBP	81 (54%) 40	G1 (*n* = 38): BSP G2 (*n* = 35): NI	PT; PH; chiropractors	13	G1: 20 sessions (60′)	VAS; general LBP function; number of LBP episodes; days of sick leave	G1 significantly improved in pain, functionality, number of episodes, and days of sick leave versus G2.
Meng et al. (2009) [40]	NLBP	360 (64%) 49	G1 (*n* = 175): BSP G2 (*n* = 159): ET	PT; PH; PSC	(*Not described*)	G1: 6 sessions (60′) + 1 session (30′) G1: 6 sessions (60′) + 1 session (30′)	NRS; FFbH; FABQ; SF-12	G1 significantly improved pain and fear versus G2. There were no significant differences between groups in quality of life and functionality.
Morone et al. (2012) [43]	NLBP	75 (72%) 55	G1 (*n* = 25): BSP G2 (*n* = 25): Perceptive rehabilitation G3 (*n* = 25): NI	PT; PH	4	G1: 10 sessions (45′) G2: 12 sessions (45′)	VAS; MPQ; ODI; WDI	There was a significant enhancement in all results for both groups from the initial stage to the point after the intervention. G2 significantly improved in pain versus G3 and G1. There were no significant differences in disability from baseline to post-intervention, nor between groups.
Morone et al. (2011) [42]	NLBP	70 (64%) 60	G1 (*n* = 41): BSP G2 (*n* = 29): NI	PT	4	10 sessions (60′)	VAS; ODI; WDI; SF-36	There was a significant enhancement in all results for both groups from the initial stage to the point after the intervention. G1 significantly improved in pain versus G2. G1 significantly improved in disability from baseline to post-intervention. But there were no significant differences between groups. No significant differences were found in quality of life.
Pakbaz et al. (2019) [44]	NLBP	64 (75%) 39	G1 (*n* = 32): BSP G2 (*n* = 32): HE	PT	1	1 session (180′)	VAS; RMDQ	There was a significant enhancement in pain and disability for G1 versus G2 from the initial stage to the point after the intervention.
Paolucci et al. (2017) [46]	NLBP	53 (82%) 61	G1 (*n* = 27): BSP G2 (*n* = 26): Feldenkrais	PT; PH	5	G1: 10 sessions (60′) G2: 10 sessions (60′)	VAS; MPQ; WDI; SF-36; MAIA	Both groups significantly improved in pain, disability, interoceptive awareness, physical role, and general and mental health from baseline to post-intervention.
Paolucci et al. (2012) [45]	NLBP	30 (Not described) 59	G1 (*n* = 15): BSP G2 (*n* = 15): Perceptive rehabilitation	PT	4	G1: 10 sessions (45′) G2: 12 sessions (45′)	MPQ; stabilimeter	There were no significant differences in pain between groups. G1 significantly improved anteroposterior velocity in stabilimeter with eyes open from baseline to post-intervention. G2 significantly improved laterolateral velocity and sway length in stabilimeter with eyes open from baseline to post-intervention. Neither G1 nor G2 significantly improved any other stabilimeter components.
Vollenbroek-Hutten et al. (2004) [47]	NLBP	163 (Not described) 39	G1 (*n* = 73): BSP G2 (*n* = 79): UC	PT; PH; PSC; trainer; dietician	7	G1: weekly ET (180′) + swimming (30′) + occupational rehabilitation (90′) + physiotherapy (240′)	RMDQ; EuroQol-5D	G1 significantly improved pain and disability versus G2.
Weber et al. (1996) [48]	NLBP	1365 (80.5%) Not described	G1 (*n* = 494): BSP G2 (*n* = 371): NI	PT	8	G1: 8 sessions (90′)	Pain incidence medical visit; drug intake	G1 significantly improved points of pain and medical visits versus G2. Both groups significantly improved drug intake at post-intervention. Neither group significantly improved pain intensity.

NLBP: non-specific low back pain; G1: group 1; BSP: Back School program; G2; group 2; NI: no intervention; PT: physiotherapist; VAS: Visual Analogue Scale; RMDQ: Roland Morris Disability Questionnaire; G3: group 3; PH: physician; PRS: Roland Morris Pain Rating Scale; SF-36: Short Form 36 Health Survey; ET: exercise therapy; NRS: numeric rating scale; GPE: global perceived effect; PSFS: Patient-Specific Functioning Scale; SPP: subjective pain perception; ODI: Oswestry Disability Index; 6MWT: 6 min walking test; BDI: Beck Depression Inventory scores; FFD: finger-to-floor distance; TFMS: trunk flexor muscle strength; TEMS; trunk extensor muscle strength; QMS: quadricep muscle strength; ROM: range of motion; WHOQOL-BREF: World Health Organization Quality of Life–BREF instrument; NNP: non-specific neck pain; EuroQol-5D: EuroQualityofLife-5D; NDI: Neck Disability Index; FFbH: Hannover Functional Ability Questionnaire; UC: usual care; TSK: Tampa Scale of Kinesiophobia; LKQ: Low Back Pain Knowledge Questionnaire; GPAQ: Activity Questionnaire; MFCS: Mean Functional Capacity Score; PSC: psychologist; FABQ: Fear Avoidance Beliefs Questionnaire; SF-12: Short Form 12 Health Survey; MPQ: McGill Pain Questionnaire; WDI: Waddel Disability Index; HE: health education; MAIA: Multidimensional Assessment of Interoceptive Awareness Questionnaire.

## Data Availability

The data that support the findings of this study are available upon request from the corresponding author, P.H.-L.

## Supplementary Materials

The following supporting information can be downloaded at https://www.mdpi.com/article/10.3390/jpm14030272/s1, Table S1: Search strategy according to the focused question (PICO); Table S2: Characteristics of the interventions [24,25,26,27,28,29,30,31,32,33,34,35,36,37,38,39,40,41,42,43,44,45,46,47,48].
