# Supplementary material for: The Effects of Back Schools on Non-Specific Back Pain: A Systematic Review and Meta-Analysis
Source: J Pers Med. 2024 Feb 29;14(3):272. doi: 10.3390/jpm14030272 (PMC10971154; doi:10.3390/jpm14030272)
Supplement: Supplementary file 1 [file jpm-14-00272-s001.zip › Supplementary Material Table S2.pdf]

Table S2. Characteristics of the interventions.

Author	Groups (Sample)	BSP	Control group
Andrade et al. (2008) [24]	G1 (n=29): BSP<br>G2 (n=28): NI	Four 60-minute weekly lessons of the "Spine School" based on Forssell's original program. In the theoretical session, an explanation of the anatomy and function of the spine, and the correct way to lie down and sleep was made. The practical sessions consisted of stretching exercises of the paravertebral musculature, strengthening of the extensor musculature of the hip, strengthening of the flexor musculature of the hip, and strengthening of the abdominal muscles. Three sets of 8 to 12 repetitions were performed for all strengthening exercises. At the end of the class, all patients received an informative booklet about exercises and postures suitable for all types of daily activities.	Waiting list
Cecchi et al. (2010) [25]	G1 (n=68): BSP<br>G2 (n=68): Individual physiotherapy<br>G3 (n=69): Spine manipulation	15 one-hour sessions, 5 days a week. In the theoretical sessions, information and group discussions on back physiology and pathology, with reassurance on the benign character of common low back pain, and with education in ergonomics at home and in different occupational settings were taught. The practical sessions included relaxation techniques, postural and respiratory group exercises, and individually tailored back exercises.	G2: 15 60-minute sessions, five times a week, for three consecutive weeks. Individual physiotherapy included passive and assisted mobilization, active exercise, 2 massage/treatment of the soft tissues, and proprioceptive neuromuscular facilitation, with emphasis on patient education and active treatment.<br>G3: 4–6 weekly 20-minute sessions for 4–6 weeks of treatment. The whole spine was examined by static and dynamic assessment. Treatment was aimed at restoring the physiological movement in the dysfunctional vertebral segments and consisted in vertebral direct and indirect mobilization and manipulation.
Costantino et al. (2014) [26]	G1 (n=27): BSP<br>G2 (n=27): Hydrotherapy	12 one-hour sessions, twice a week. In the theoretical sessions individuals were informed about the anatomy of spinal column, its functioning and ergonomic position and the basis of the pain-inducing mechanism, psychological aspects and stress management. The practical sessions included stretching and muscular strengthening, associated with proper breathing.	Participants at first performed walking exercises to adapt to the pool conditions, afterwards they performed bilateral stretching and selective muscle strengthening exercises
Devasahayam et al. (2014) [27]	G1 (n=9): BSP<br>G2 (n=6): ET	One-hour session. In the theoretical section, participants were taught about correct postures like upright sitting and standing postures, proper body mechanics of ADLs like lifting, mopping and sweeping, walking, going up and down the stairs, information on ergonomic correction and activity pacing. The practical section included functional exercises	Participants group performed generic mat-based exercises commonly used to treat chronic NSLBP. These exercises were not focused on any specific body mechanics or postures. They performed stretching exercises (quadriceps, hamstrings, calf, hip external rotators and for the spine), mat exercises (knee hugs, knee rocking, lumbar rotation and pelvic tilts in the supine position), and mat-based core stability exercises (crook lying, alternate upper and lower limb movements in prone and four-point kneel

		and stretching exercises in an upright standing posture were performed. In the CBT section participants were asked to report difficult tasks in their daily functional activities and rate them in a patient-specific functional score form. These tasks were noted and were emphasized during the functional task practice time.	position, double leg and single leg bridging, overhead arm flexion movements in crook lying, hundreds, dead bugs, forward and sideward planks)
Donchin et al. (1990) [28]	G1 (n=46): BSP G2 (n=46): Calisthenics for the back G3 (n=50): NI	Four 90-minute sessions twice a week, plus a fifth session after 2 months. In the theoretical sessions, participants were given instruction in proper body mechanics as well in exercises for the back and abdominal muscle. In the practical sessions participants were encouraged to exercise at home on their own.	G2: 45-minute sessions, biweekly, for three months. The session included flexion and pelvic tilt exercises, based on the Williams method, aimed at strengthening the abdominal muscles, expanding spinal forward flexion, and rectifying the general posture. G3: Waiting list.
Donzelli et al. (2006) [29]	G1 (n=22): BSP G2 (n=21): Pilates	10 consecutive one-hour sessions. In the theoretical sessions, participants were taught theoretical notions in the anatomy and pathology of the spinal column and in the principles of postural education. The practical sessions postural included education exercises, respiratory education, muscular extension and strengthening exercises of the paravertebral muscles and lower limbs, mobilizing exercises for the spinal column and antalgic postures.	10 consecutive one-hour sessions. The Pilates protocol comprised 9 modules: postural education, search for neutral position, sitting exercises, antalgic exercises, stretching exercises, proprioceptively improvement exercises, breathing education, mobilization of the cervical rachis and the scapula-humeral joint, and theoretical explanations. As was done in the Back School group, the physiotherapist explained the exercise rationale during the treatment session. In the practical session, the basic level exercises of the Pilates CovaTech "Mat4me" program were performed.
Durmus et al. (2014) [30]	G1 (n=61): BSP G2 (n=60): ET	Eight one-hour sessions in two months. In the theoretical sessions, the patients were taught about the functional anatomy of the back- low back, the correct use of the lower back, reasons for the pain, skills to enable them to cope with low back problems and increase self-esteem. In the practical sessions, patients practiced How to pick up and carry objects, how to lie down in the bed, how to get up from the bed, how to use home utensils. In general, how to protect low back in daily life. The exercise part lasted three weekly one-hour-sessions for three months. It consisted of 4 exercises: 1. Motion, flexibility, and back strengthening exercises of the cervical, thoracic, and lumbar spine; stretching of the erector spine muscle, hamstring muscles, pelvic muscles, and abdominal muscles 2. Special exercises to correct mobility of the spine and hip joints, activate the stabilizing muscles of the spine, and increase flexibility of the lower limb muscles. 3. Functional exercises to improve postural control, dynamic	The only exercise program lasted three weekly one-hour-sessions for three months. It consisted of 4 exercises: 1. Motion, flexibility, and back strengthening exercises of the cervical, thoracic, and lumbar spine; stretching of the erector spine muscle, hamstring muscles, pelvic muscles, and abdominal muscles 2. Special exercises to correct mobility of the spine and hip joints, activate the stabilizing muscles of the spine, and increase flexibility of the lower limb muscles. 3. Functional exercises to improve postural control, dynamic body balance, and coordination.4. Progressive relaxation exercises to normalize muscle tension.

		body balance, and coordination.4. Progressive relaxation exercises to normalize muscle tension.	
García et al. (2013) [31]	G1 (n=72): BSP<br>G2 (n=74): McKenzie	Four weekly one-hour sessions in four weeks. In the theoretical sessions, participants were given advice that included basic components of anatomy and biomechanics of the spine, ideal posture and rest postures, ergonomics, and most common types of treatment. In the practical sessions, participants performed exercises that aimed to improve mobility, flexibility, and strength.	Four weekly one-hour sessions in four weeks. Patients allocated to the McKenzie group received theoretical information regarding the care of the spine and performed specific exercises according to the direction of preference of movement identified by a relief of pain, centralization of the pain, or abolishment of pain.
Henkel et al. (2009) [32]	G1 (n=27): BSP<br>G2 (n=28): Nordic Walking<br>G3 (n=30): Barefoot Walking	24 bi-weekly sessions for 12 weeks. Not described.	G2: 24 bi-weekly sessions for 12 weeks. Not described.<br>G3: 12 weekly sessions for 12 weeks. Not described.
Heymans et al. (2006) [33]	G1 (n=98): BSP high intensity<br>G2 (n=98): BSP low intensity<br>G3 (n=103): UC	16 bi-weekly one-hour sessions in eight weeks. In the theoretical sessions, the physiotherapist promoted a time contingent increase in the level of activity. Principles of CBT were applied throughout the back school program. In the practical sessions, participants performed individual exercises simulating the activities the worker experienced as the most problematic at the workplace, work-simulating and strength training exercises were performed with gradually increasing resistance. The workers were also given home exercises during the time they were participating in the back school program.	Four weekly two-hour sessions. In the theoretical sessions, the physiotherapist discussed the workplace situation. Not only the most problematic activities experienced by the worker because of the low back pain will be discussed, workers also received information on how to cope with these activities. In the practical sessions, participants performed a standardized exercise program consisting of strength training and home exercises. The strength training involved progressive resistance training as well as functional exercises. Workers were instructed to perform exercises at home twice a day.
Hurri et al. (1989) [34]	G1 (n=93): BSP<br>G2 (n=92): NI	Six bi-weekly one-hour sessions. Patients in the experimental group attended education and exercise sessions.	15-page pamphlet focused on basic anatomy and physiology of the spine, principles of ergonomics for low back pain patients, instructions on how to exercise the body muscles and how to cope with the acute phase of low back pain. No actual treatment was administered to the control group.
Ibrahim et al. (2020) [35]	G1 (n=140): BSP<br>G2 (n=141): UC	13 two-hour sessions. The theoretical sessions were organized in four sections: Section 1 consists of education on the structure and function of the backbone, Section 2 consisted of education on LBP and work-related back pain, Section three consisted of education on methods of preventing LBP among nurses, and Section 4 consisted of an exercise method to address LBP among nurses. In the practical sessions, participants performed exercises aimed at improving mobility, flexibility, and strength. With the addition of a gym ball exercise, the intervention package developed for this study consisted of exercises for	The control group was given standard care and advice for a sedentary lifestyle by an attending a doctor without referring to any specific module.

		stretching, strengthening, mobilizing, and exercising core stability.	
Ibrahimi et al. (2022) [36]	G1 (n=125): BSP<br>G2 (n=125): NI	Four two-hour sessions. In the theoretical sessions, participants received anatomy and biomechanics of the spine, epidemiology of LBP and musculoskeletal disorders, principles of body biomechanics variation of the mechanical forces in different movements of the back, determining awkward postures, theoretical presentation of relaxation posture, providing proper postures, guidance on position when seated or standing or handling manual loads, instruction on breathing exercises, kinesthetic training, stretching of the lower back, quadriceps, and hamstring muscles. In the practical sessions, participants exercise for abdominal muscular strength, orientation about joint protection during daily activities.	LBP management guide booklet.
Jaromi et al. (2012) [37]	G1 (n=56): BSP<br>G2 (n=55): Passive physiotherapy	Six weekly 50-minute sessions. In the theoretical sessions, participants received relevant education in anatomy and body mechanics, biomechanics, biomechanical risk factor and ergonomics theory. In the practical sessions, participants performed stretching muscles that are prone to shrinkage and hypertonic, strengthening muscles that are prone to weakening and hypotonic.	Six weekly sessions. The passive physiotherapy group received transcutaneous electrical nerve stimulation therapy and heat therapy
Klaber-Moffett et al. (1986) [41]	G1 (n=40): BSP<br>G2 (n=38): ET	Three sessions. In the theoretical sessions, participants anatomy and biomechanics of the spine and common causes of low back pain were taught. In the practical sessions, participants performed exercises aimed particularly at strengthening the abdominal muscles.	Three sessions. In the only exercise group, participants performed exercises aimed particularly at strengthening the abdominal muscles.
Lankhorst et al. (1983) [38]	G1 (n=21): BSP<br>G2 (n=22): Electrotherapy	Four bi-weekly sessions of the Back School	Four bi-weekly sessions of detuned pulsating shortwave applications.
Lønn et al. (1999) [39]	G1 (n=38): BSP<br>G2 (n=35): NI	20 one-hour sessions. In the theoretical sessions, participants received anatomy, biomechanics, pathology, and basic ergonomic principle related to the spinal column and pelvis. In the practical sessions, participants applying the basic ergonomic principles, which included bending the knee and hip joints while keeping the lumbar segments near mid position and using short lever arms during functional exercises and obstacle course simulations of home and work environments such as vacuum cleaning, floor washing, and lifting maneuvers; strength training of leg	Waiting list.

		muscles and muscles between the upper body and pelvis was given too.	
Meng et al. (2009) [40]	G1 (n=175): BSP<br>G2 (n=159): ET	Six one-hour sessions plus one 30-minute session. In the theoretical sessions, participants received information about epidemiology, causes, course, risk factors and therapy methods, structure and function of the spine, body perception, movement behavior and pain or health body perception during movement sequences, muscle activation strategies to stabilize the spine, pain experience, avoidance, and persistence behaviors, coping strategies. In the practical sessions, participants performed exercises about sitting, bending, lifting, carrying for spinal stabilization at home and work.	Six one-hour sessions plus one 30-minute session. Usual care.
Morone et al. (2012) [43]	G1 (n=25): BSP<br>G2 (n=25): Perceptive rehabilitation<br>G3 (n=25): NI	10 45-minute sessions in four weeks. In the theoretical sessions, participants received general anatomical information on the spine, its function and ergonomic positions in daily living were explained. In the practical sessions, participants performed exercises, based on the re-education of breathing, self-stretching of the trunk muscles, erector spine reinforcement, abdominal reinforcement, and postural exercises.	G2: Perceptive Rehabilitation was developed as a therapeutic system, based on the interaction between the patient's body surface and a support surface, comprising small latex cones, varying in dimension, and elasticity. The cones are applied with their inferior bases on a rigid wood surface through elastic strips.<br>G3: control group comprised participants who received only medical and pharmacological assistance, such as analgesics, myorelaxants, and nonsteroidal anti-inflammatory drugs, as needed and under medical supervision.
Morone et al. (2011) [42]	G1 (n=41): BSP<br>G2 (n=29): NI	10 one-hour sessions in four weeks. In the theoretical sessions, participants received general anatomical knowledge of spine, its function and ergonomic positions in daily living, information about pain concepts, psychological aspects and stress management, workplace situation and sport activities. In the practical sessions, participants performed exercises based on the re-education of breathing, self-stretching trunk muscles, erector spine reinforcement, abdominal reinforcement, and postural exercises.	Control group comprised participants receiving only medical/pharmacological assistance. They used drugs like analgesics, myorelaxants and nonsteroidal anti-inflammatory drugs when needed and under medical supervision.
Pakbaz et al. (2019) [44]	G1 (n=32): BSP<br>G2 (n=32): HE	One three-hour sessions. In the theoretical sessions, participants received information on the structure and function of the spine, biomechanics, optimal posture, and ergonomics. In the practical sessions, participants performed special back exercises in accordance with Back School program.	Waiting list. After the end of the study, they received a booklet about LBP.
Paolucci et al. (2017) [46]	G1 (n=27): BSP	10 bi-weekly one-hour sessions. In the theoretical sessions, participants received general anatomical information on the	10 bi-weekly one-hour sessions. The Feldenkrais method encouraged the exploration of movements that were related to a specific function to

	G2 (n=26): Feldenkrais	spine and its function and ergonomic positions in daily living, information on chronic pain and LBP, the related psychological aspects, and stress management. In the practical sessions, participants performed exercises that were based on diaphragmatic breathing, self-stretching the trunk muscles, erector spine reinforcement, abdominal reinforcement, and postural exercises.	enhance one's awareness of how movements are performed and to invite the participant to investigate how he can expand his actions and functional abilities. The lessons addressed habitual patterns of movement and sought to expand a person's self-image.
Paolucci et al. (2012) [45]	G1 (n=15): BSP G2 (n=15): Perceptive rehabilitation	10 45-minute sessions in four weeks. In the theoretical sessions, participants received general anatomical information on the spine, its function, and ergonomic positions in daily living. In the practical sessions, participants performed exercises, based on re-education of breathing, self-stretching of the trunk muscles, erector spine reinforcement, abdominal reinforcement, and postural exercises.	12 45-minute sessions three times a week. The Perceptive group received rehabilitation with a specific tool called surface for perceptive rehabilitation', this tool is developed as a therapeutic system, based on the interaction between the patient's body trunk and a support surface. It comprises about 100 deformable latex cones with a small top, fixed to a rigid surface. The cones vary in dimensions and elasticity.
Vollenbroek-Hutten et al. (2004) [47]	G1 (n=73): BSP G2 (n=79): UC	Three hours of conditional training and sport, 30 minutes of swimming, 90 minutes of occupational therapy and 4 hours of physiotherapy each week for seven weeks. In the theoretical sessions, participants received information about learning skills to make optimal use of the remaining physical capabilities. In the practical sessions, participants performed conditional training and sport, occupational therapy, and physiotherapy.	The usual care group did not receive any treatment at the rehabilitation center, but they were allowed to apply for usual health care facilities outside the rehabilitation center.
Weber et al. (1996) [48]	G1 (n=494): BSP G2 (n=371): NI	Eight weekly90-minute sessions. In the theoretical sessions, participants received theoretical information on epidemiology, anatomy, biomechanics, diagnostic tools, pain concepts, psychological aspects, and stress management. In the practical sessions, participants performed exercises about ergonomics, workplace evaluation, muscle physiology, strength, fitness, sport activities and self-care.	Waiting list.

G1: group 1; BSP: Back School Program; G2; group 2; NI: no intervention; G3: group 3; ET: exercise therapy; ADLs: activities of daily life; CBT: cognitive behavioral therapy; NSLBP: Non-Specific Low Back Pain; UC: usual care; LBP: low back pain; HE: health education.
