# Supplementary material for: The Effects of Back Schools on Non-Specific Back Pain: A Systematic Review and Meta-Analysis
Source: J Pers Med. 2024 Feb 29;14(3):272. doi: 10.3390/jpm14030272 (PMC10971154; doi:10.3390/jpm14030272)
Supplement: Supplementary file 1 [file jpm-14-00272-s001.zip › Supplementary Material Table S1.pdf]

**Table S1.** Search strategy according to the focused question (PICO).

| Database | Search equation |
|---|---|
| PubMed | (((("Back Pain"[Mesh]) OR "Low Back Pain"[Mesh]) OR "Neck Pain"[Mesh]) OR "Musculoskeletal Pain"[Mesh]) AND "back school" |
| Web of Science | TS= (("Back Pain" OR "Low Back Pain" OR "Neck Pain" OR "Musculoskeletal Pain") AND ("back school")) |
| Scopus | TITLE-ABS-KEY ((“back pain" OR "low back pain" OR "neck pain" OR "musculoskeletal pain”) AND (“back school" )) |
| Medline | MH ((“back pain" OR "low back pain" OR "neck pain" OR "musculoskeletal pain”)) AND (“back school") |
